# Meeting the educational needs of children with hearing loss

**DOI:** 10.2471/BLT.18.227561

**Published:** 2019-09-03

**Authors:** Karissa L LeClair, James E Saunders

**Affiliations:** a Geisel School of Medicine, Dartmouth College, Hanover, New Hampshire, United States of America (USA).; b Department of Otolaryngology, Dartmouth-Hitchcock Medical Center, 1 Medical Center Drive, Lebanon, New Hampshire 03756, USA.

Paediatric hearing loss is a growing public health issue that is currently a significant barrier to achieving sustainable development goal 4 (SDG 4), that is, quality education for all.^1^ When children with hearing loss do not receive treatment, they might have difficulty accessing mainstream schooling and therefore obtain worse educational outcomes. The severity of these implications is correlated with level of hearing loss and earlier age of onset.^2^^,^^3^ Children with any degree of hearing impairment have been shown to exhibit poor language development, leading to lower literacy rates, diminished social skills and impaired executive function capacity.^3^ Different severities of hearing loss must be considered, as a child’s ability to access schooling and participate in integrated education is highly dependent on level of hearing loss.^4^ A child’s educational ability can be affected at a milder severity of hearing loss than what was considered as disabling. Consequently, the global burden of educationally disabling hearing loss is larger than previously estimated by the World Health Organization (WHO).^5^

Mild hearing loss, that is, a hearing level threshold between 26–40 decibel (dB) in the better ear, affects almost 50 million children worldwide, yet is frequently overlooked and undertreated.^6^ A 2016 review concluded that children with mild hearing loss, overall, tend to have compromised speech recognition and poorer language skills.^7^ Therefore, these children are over 2.5 times more likely to have academic difficulties and they more commonly experience grade retention. One study indicated that 37% (24/66) of children in a cohort with mild hearing loss had failed at least one grade.^7^^,^^8^ With respect to children affected by moderate hearing loss (41–60 dB), educational impairments mirror the deficits of those with mild losses, yet are more common and impactful. A study from France reported nearly half of children with moderate hearing loss had experienced one or more years of grade retention.^8^ Standardized academic test scores for children at all levels of hearing impairment are significantly lower than those of children with normal hearing, and notably demonstrate a strong correlation with the severity of hearing loss.^2^

Academic achievement for children with severe (61–80 dB) to profound (> 80 dB) hearing loss is significantly hindered relative to peers, with one third of children functionally illiterate upon graduation from secondary school.^9^ Furthermore, severe to profound hearing loss has been shown to have a significant effect on a child’s ability to participate in mainstream education. A study in the United States of America showed that only 22% (1536/6980) of children with severe hearing loss and 10% (1517/15 174) of those with profound hearing loss participated in integrated mainstream education for more than half of their school day.^4^ These data identify a crucial objective for improvement, as those with hearing loss who are unable to access mainstream education face impaired academic and language outcomes.^2^

Children with unilateral hearing loss (that is, who are hearing impaired in one ear only) should also be included, as they may experience similar educational barriers as children with mild deficits. Unilateral hearing loss causes difficulty in localization of sound and impairs the ability to hear in noisy settings such as a classroom. Additionally, this deficit has been associated with lower oral language scores when compared to normal-hearing counterparts.^7^

The global prevalence of hearing loss must be discussed to better understand the burden of this condition. Research has traditionally focused on characterizing the prevalence of disabling hearing loss (> 30 dB) – identifying 34 million children worldwide affected at this threshold or greater.^5^ However, even mild hearing loss has potential for detrimental effects on education.^7^^,^^8^ Therefore, educationally disabling hearing loss should include all thresholds of mild hearing loss, significantly increasing the number of children characterized as at risk. To calculate updated numbers of children affected at each level of disability, global prevalence rates were applied to the most recent 2018 population data.^6^ These estimates identify 61 million children from birth to 14 years of age, globally, who have some degree of hearing impairment with potential effect on their educational outcomes. This number nearly doubles the previous estimate of children whose hearing loss met thresholds traditionally considered disabling.^5^^,^^6^

Latest available global prevalence estimates of childhood hearing loss range show significant regional variability. Low- and middle-income countries in geographical areas, such as south Asia, are disparately affected: 82.5% (95% confidence interval, CI: 45–151.4) for mild hearing loss; 14.3% (95% CI: 7.4–29.7) for moderate hearing loss; 0.7% (95% CI: 0.3–1.4) for severe hearing loss; and 0.2% (95% CI: 0.1–0.4) for profound hearing loss, while high-income countries contribute only a small fraction of the global burden. In these countries, the prevalence for mild hearing loss is 17% (95% CI: 12.4–25); for moderate hearing loss is 2.7% (95% CI: 2.0–4.0); for severe hearing loss is 0.1% (95% CI: 0.1–0.2); and for profound hearing loss is 0.0% (95% CI: 0.0–0.1).^6^ Rates of disabling hearing loss have been shown to increase exponentially as gross national income decreases, with a nearly fivefold prevalence in some low- and middle-income regions as compared to high-income nations.^6^ Following this same trend, access to hearing health care is correlated with national income levels. Low- and middle-income countries consistently report insufficient numbers of otolaryngologists, audiologists and speech therapists per capita.^10^ While WHO’s minimum standard recommends 40 otolaryngologists per 1 million people, more than two thirds of low-income countries do not even have one otolaryngologist per million people.^10^ With respect to device availability, global production of hearing aids is estimated to meet less than 10% of global need and less than 3% of need in low-income countries.^5^

While the current global state of paediatric hearing loss may appear dismal, promising data indicates that 60% of this hearing loss is preventable. These common preventable causes include infectious causes, birth complications, noise exposure and the use of ototoxic medications in pregnant women and children.^5^ Notably, WHO estimates that preventable causes are more likely to be the etiology of hearing loss in low- and middle-income countries (75% of all hearing loss) as compared to high-income countries (49%).^5^ This disparity has been attributed to regional trends such as higher infection rates, overuse of ototoxic antibiotics, and lack of adequate maternal and fetal care in low- and middle-income countries.^5^ In addition to the opportunity for primary prevention, evidence demonstrates that early detection and treatment can protect against language and educational consequences.^3^ Even children with profound hearing loss exhibited speech and language levels equal to their hearing peers after receiving early diagnosis and a treatment regime of hearing amplification, auditory and verbal therapy, and cochlear implantation by 18 months of age.^11^ The specific intervention must be tailored to the level of hearing loss and in-country resources, but promisingly, several effective options including hearing aids, speech rehabilitation, cochlear implantation and deaf education are available.^5^ Newborn hearing screening is a crucial aspect of this process, as early detection leads to early intervention, a vital predictor of future language and educational outcomes.^3^

To assess global progress towards achieving quality education for all, evidence of paediatric hearing loss being a barrier to this goal should be considered. SDG 4 designates universal education as one of our highest global priorities, specifically aiming to ensure equal access to all levels of education for the vulnerable, including persons with disabilities.^1^ To achieve this quality education mandate, we must first address the educational repercussions of this common disability through appropriate efforts in hearing loss prevention, identification and treatment.

Particularly within low- and middle-income countries, increased preventative efforts will be the most cost–effective and high-yield strategy to combat educationally disabling hearing loss. Reduction of paediatric hearing loss can be accomplished through expansion of basic low-cost health provisions, such as measles, mumps and rubella vaccines to prevent rubella-associated hearing loss. Another option is to provide easily accessible treatment for otitis media, to address chronic ear infections as a common etiology. Awareness raising will be the single most important aspect of addressing paediatric hearing loss on a global scale. Patients and health providers must be informed on topics such as the ototoxic effects of unregulated antibiotic use, safe and hygienic practices for labour and delivery, and potentially-dangerous exposures for mothers during pregnancy. However, education should also include expanding awareness of the widespread burden of paediatric hearing loss, highlighting common early presentations of this disease and emphasizing the potential for long-term effects if left untreated. Such awareness will be crucial in fighting the stigma associated to this disability, both encouraging schools to provide resources for inclusion of affected children, as well as enabling parents to recognize and seek treatment for children at an early age.

Beyond prevention, newborn screening for early identification of hearing loss should be expanded and standardized. However, such efforts will require sufficient availability of otolaryngology and audiology resources to provide treatment following detection. This objective will require hearing health-care investment and expansion in low- and middle-income countries, where children currently have high rates of hearing loss, yet have little or no access to rehabilitation services. Although this objective would be a large-scale investment, the long-term outcomes of hearing loss detection and intervention programmes have been shown to be cost–effective because they mitigate educational deficits and lost productivity.^5^ Children with mild hearing loss must also be included when considering the scope of such investment, as even mild deficits are frequently detrimental to a child’s language ability and educational access. If the global community hopes to achieve SDG 4, a concrete objective is to first address this treatable disability.
